# A cross-sectional study of knowledge about secondhand smoke-attributed diseases, awareness, and applicability of the smoking control law in a southern province of Thailand

**DOI:** 10.18332/tid/204397

**Published:** 2025-05-29

**Authors:** Chutarat Sathirapanya, Napakkawat Buathong, Polathep Vichitkunakorn, Phoomjai Sornsenee, Vasin Pipattanachat, Pornchai Sathirapanya

**Affiliations:** 1 Department of Family and Preventive Medicine, Faculty of Medicine, Prince of Songkla University, Hat Yai, Thailand; 2 Research Center for Kids and Youth Development, Prince of Songkla University, Hat Yai, Thailand; 3 Tobacco Control Research and Knowledge Management Center, Faculty of Medicine Ramathibodi Hospital, Mahidol University, Bangkok, Thailand; 4 Department of Medicine, Faculty of Medicine, Prince of Songkla University, Hat Yai, Thailand

**Keywords:** tobacco smoke, law, secondhand smoke, smoking regulation

## Abstract

**INTRODUCTION:**

Secondhand smoke (SHS) exposure is a significant cause of illness. This study aimed to explore the awareness of SHS-attributed illnesses and the legal control of the Tobacco Product Control (TPC) Act 2017 among the local people living in Meung district, a municipality of Songkhla Province, Thailand.

**METHODS:**

This quantitative and qualitative study was conducted in 3 marketplaces and 6 public bus terminals in a southern province of Thailand between November 2021 and September 2022. A total of 330 volunteers were enrolled for the quantitative analysis. Meanwhile, 13 local government officers (LGOs), 2 market visitors, and 5 public vehicle passengers were interviewed for qualitative analysis to evaluate applicability of the law in this province. We collected the participants’ general demographics, prevalence of secondhand smoke (SHS) exposure, knowledge about SHS-attributed diseases, and awareness of the law. Chi-squared test was used to assess the associations between demographics and knowledge of SHS-attributed diseases and awareness of the legal restrictions regarding SHS exposure (p<0.05). Thematic analysis for evaluating applicability of the law was conducted from the interviews.

**RESULTS:**

Tobacco smell was experienced more frequently than witnessing smokers in marketplaces (49.8% vs 30.0%) and on public vehicles (45.5% vs 20.7%). The mean ± SD scores of knowledge regarding the law were low (marketplace, 4.09 ± 1.61; bus, 4.07 ± 1.69), while that of SHS-attributed health harms were moderate (marketplace, 6.31 ± 2.14; bus, 6.30 ± 1.64). Age, education level, and religion had significant associations with knowledge about SHS-attributed diseases (p=0.001, <0.001, <0.001, respectively), while age and education level were significantly associated with awareness of the law (p<0.001). We found weaknesses in the collaboration of LGOs. Inadequate resources and a high volume of routine workload were the attributed barriers.

**CONCLUSIONS:**

Enhancing knowledge about SHS-attributed illnesses, awareness of the SHS control law, and strengthening public engagement are crucial for SHS exposure control. The collaboration between the local people and LGOs for effective SHS control is advocated.

## INTRODUCTION

The International Agency for Research on Cancer considers secondhand smoke (SHS) a ‘group 1’ carcinogen or ‘known human carcinogen’ because several carcinogenic compounds are found in the burning of tobacco or cigarettes^1^. Therefore, WHO endorsed the Framework Convention on Tobacco Control (FCTC) in 2003. This international agreement was reached in order to achieve a 30% relative reduction of smoking prevalence by the year 2025 globally^2,3^.

In Thailand, the smoking-attributed illnesses’ share of national healthcare costs was US$ 265.97 million in 2017, of which 7% was spent on medical care for non-smokers exposed to SHS^4^. Several anti-smoking campaigns have been underway since 1985, spearheaded by a non-governmental organization called the ‘Action on Smoking and Health Foundation, Thailand (ASHT)’, with the aim of lowering smoking prevalence while protecting non-smokers from SHS exposure^5^. The actions of the foundation resulted in the decrease of smoking prevalence from 43.7% to 34.7% in males and from 2.6% to 1.3% in females between 2004 and 2021^6^. Even though the Tobacco Product Control (TPC) Act legislated in 2017 permits legal action and the ASHT’s campaigns encourage smoking control, the magnitude of SHS exposure to non-smokers in public places in Thailand has not been effectively reduced.

Data from the National Statistical Office of Thailand in 2017 reported that Southern Thailand had the highest percentage of smokers in the country. Also, every province in the 12th healthcare administrative region in Southern Thailand, including Songkhla Province, had a higher average percentage of smokers (19.1%) than that of the nation (17.0%). Songkhla was notably one of the 4 provinces with the highest percentage of smokers in this region. Moreover, witnessing smokers, experiencing the smell of tobacco, or observing cigarette butts in public places were reported by more than 80% of respondents on the annual health survey of the province^7^. Furthermore, a national survey in Thailand in the same year supported the higher percentage of SHS exposure in public places, such as fresh-food markets, restaurants, and bars etc. (68.3%), than in households (32.7%)^[Bibr CIT0008]8^.

Songkhla Province is a well-known commercial and tourism hub in the lower south of Thailand. Although modern facilities such as air-conditioned supermarkets, coffee shops, convenience stores, and buses, where smoking is strictly forbidden, are available, local open-air fresh-food markets, vans, high-roofed pickup truck taxis (called ‘songthaew’ in Thai), or motorcycle taxis are all still used by a significant portion of the local people. SHS was commonly experienced but under-recognized in these places, despite the TPC Act including these spaces as smoking-free areas.

This study aimed to explore the awareness of SHS-attributed illnesses and the legal control of the TPC Act among the local people living in Meung district, a municipality of Songkhla Province, Thailand.

## METHODS

### Study design and setting

This cross-sectional quantitative study was conducted in a group of local people, and qualitative study in the local governmental offices (LGOs) in Songkhla Province of Southern Thailand. Three fresh-food marketplaces and 1 inter-provincial bus terminal and 6 local van or high-roofed pickup truck taxi (‘songthaew’) terminals in Meung district, were purposively selected. SHS exposure to the people attending these areas was common but less recognized. Also, legal restrictions on smoking in these areas were weak.

### Population and sample size

People aged ≥18 years who understood Thai well and were present at the study sites (marketplaces, bus or van terminals) as service clients were invited to participate in the study. Data collected from this group of participants underwent quantitative analysis. The sample size of this study was calculated following the principle of estimating a non-definite population proportion (n=Z^2^_α/2_ pq/d^2^; p=0.27, q=1-p, d=0.5). The total sample size required for significant statistical power was 334 (+10% for missing data).

The authorized LGOs for tobacco control in the province were enrolled purposively for in-depth interviews. The available data were analyzed qualitatively. The enrolled LGOs were the provincial health policy makers and front-line practitioners.

All participants were informed of the study details before enrollment and consent was obtained prior to performing the study.

### Collected variables

We recorded the general demographic characteristics of the local people who participated in this study, their current smoking status, percentage of visible smokers and detectable tobacco smells in the study sites, their responses to the smokers, knowledge regarding SHS control by the TPC Act 2017 and SHS-attributed health hazards, and the sources of their health information regarding smoking and SHS.

We used a designed interview questionnaire to collect the LGOs’ understanding, comments, and suggestions concerning the applicability of the TPC Act 2017, particularly the prevention of SHS exposure to non-smokers. The data obtained underwent qualitative analysis.

### Study tools

The participants’ general demographic data were recorded in case record forms. We further evaluated: 1) knowledge of the participants regarding the health hazards of SHS exposure, and 2) awareness of the legal regulations concerning smoking-free areas in public places, according to the TPC Act 2017. Each topic was composed of 10 yes-no answer questions (see Supplementary file). The results were scored one point for each correct answer and 0 points for incorrect or uncertain answers. The mean scores obtained from the individual evaluation forms were classified as: ≤6.00, low; 6.01–7.99, medium; and ≥8.00, high^9^. The evaluation questions were validated by 2 experts in public healthcare and one legal authority from the LGOs working on smoking control. The indices of item objective congruence (IOC) for content validity of the questions evaluating knowledge regarding SHS-attributed health harms and awareness of the TPC ACT 2017, were 0.93 and 0.90, respectively. The Cronbach’s alpha coefficients for reliability were 0.84 and 0.78, respectively.

We interviewed 13 LGOs involved in tobacco control actions authorized by the TPC Act 2017, 2 market visitors, and 5 bus passengers presented at the study sites regarding the feasibility, limitations, and suggestions concerning enhancing tobacco control and promoting smoking-free zones in the province. The answers to the in-depth interviews were collected for qualitative analysis (see Supplementary file).

### Statistical analysis

Numerical data are shown as frequencies (n) and percentages (%). Mean and standard deviation (SD), and median and interquartile range (IQR), were applied for continuous and categorical data, respectively. The chi-squared test was used to analyze for significant associations between the study variables (p<0.05). A thematic method was used for the analysis of qualitative data.

## RESULTS

There were 330 participants, of whom 209 and 121 were from the marketplaces (market visitors) and bus terminals (bus passengers), respectively. Most of the participants were female (80%). The market visitors had a higher mean (SD) age [51.44 years (11.92)]. The education levels between the 2 groups were comparable. Most of the participants in both groups were non-smokers (89.7%). The market visitors commonly received knowledge about the SHS-attributed health hazards through printed materials (26.8%), while 43.8% of the bus passengers received it through online media, such as ‘YouTube’ or other online channels (Table 1).

**Table 1:** Sociodemographic and smoking details of the participants present at the marketplaces and public vehicles transportation during the study period in Meung district of Songkhla Province, Thailand, November 2021– September 2022 (N=330)

*Characteristics*	*Categories*	*Market visitors* *n (%)*	*Bus passengers* *n (%)*	*Total* *n (%)*
**Total**		209 (63.3)	121 (36.7)	330 (100)
**Sex**	Male	35 (16.7)	31 (25.6)	66 (20.0)
	Female	174 (83.3)	90 (74.4)	264 (80.0)
**Age** (years), mean (SD), range		51.44 (11.92), (27–79)	24.44 (7.49), (17–51)	41.54 (16.74), (17–79)
**Education level**	Primary school	46 (22.0)	19 (15.7)	65 (19.7)
	Secondary school	97 (46.4)	60 (49.6)	157 (47.6)
	Bachelor’s	60 (28.7)	35 (28.9)	95 (28.8)
	Higher than Bachelor’s	6 (2.9)	7 (5.8)	13 (3.9)
**Religion**	Buddhism	171 (81.8)	109 (90.1)	280 (84.8)
	Islam	38 (18.2)	12 (9.9)	50 (18.2)
**Smoking status**	Never	188 (90.0)	103 (85.1)	291 (88.2)
	Ceased	3 (1.4)	0 (0)	3 (0.9)
	Daily	9 (4.3)	11 (9.1)	20 (6.1)
	Occasionally	9 (4.3)	7 (8.5)	16 (4.8)
**Types of tobacco smoked**	Cigarettes	14 (77.8)	13 (68.4)	27 (73.0)
	Roll-your-own	4 (22.2)	1 (5.3)	5 (13.5)
	Other (incl. e-cigarettes)	0 (0)	5 (26.3)	5 (13.5)
	Total	18 (100)	19 (100)	37 (100)
**Sources of tobacco information**	Television	88 (23.2)	49 (18.0)	137 (21.0)
	Radio	17 (4.5)	12 (4.4)	29 (4.5)
	Printed material	102 (26.8)	31 (11.4)	133 (20.4)
	YouTube or other online media	67 (17.6)	119 (43.8)	186 (28.5)
	Posters	78 (20.5)	42 (15.4)	120 (18.4)
	Associates	28 (7.4)	19 (7.0)	47 (7.2)

There were 49.8% and 45.5% of market visitors and bus passengers who reported the experience of tobacco smells, whereas the number of visible smokers present at the sites were only 30.0% and 20.7%, respectively. The largest proportion of visible smokers were the goods transporters in the market (52.1%) and the bus or van drivers (80.0%). The market visitors (74.6%) and bus passengers (45.5%) did not take any actions against the smokers; only 10% of market visitors and 3.3% of bus passengers intended to report the event to an authorized officer or provincial agency; however, they did not know how to undertake any such action (Table 2).

**Table 2:** Frequency of witnessing smokers, experience of tobacco smell in the marketplaces and public vehicles transportation, and the reactions against the smokers obtained from the self-reported data of the study participants in Meung district of Songkhla Province, Thailand, November 2021– September 2022 (N=330)

*Variables*	*Market visitors* *n (%)*	*Bus passengers* *n (%)*	*Total* *n (%)*
**Total**	209 (63.3)	121 (36.7)	330 (100)
**Witnessing smokers**			
No	161 (70.0)	96 (79.3)	257 (77.9)
Yes	48 (30.0)	25 (20.7)	73 (22.1)
**Smokers observed**			
Buyers	11 (22.9)		
Sellers	12 (25.0)		
Goods transporters	25 (52.1)		
Drivers		20 (80.0)	
Passengers		4 (16.0)	
Others		1 (4.0)	
**Experienced tobacco smoke smell**			
No	105 (50.2)	66 (54.5)	171 (51.8)
Yes	104 (49.8)	55 (45.5)	159 (48.2)
**Reactions**			
None	156 (74.6)	55 (45.5)	211 (63.9)
Ask the smokers to move away	24 (11.5)	52 (43.0)	76 (23.0)
Request the smokers to stop smoking	8 (3.9)	10 (8.3)	18 (5.5)
Intended to report, but did not know how to do	21 (10.0)	4 (3.3)	25 (7.6)
**Knowledge scores^§^**			
About the legislated smoking-control law	4.09 (1.61)	4.07 (1.69)	
About secondhand smoke-attributed heath harms	6.31 (2.14)	6.30 (1.64)	

§ Scores ≤6.00, low; 6.01–7.99, medium; and ≥8.00: high.

The mean ± SD scores of knowledge regarding the law on smoking-free zones in public places were low in both groups (4.09 ± 1.61, market visitors; 4.07 ± 1.69, bus passengers), while that of SHS-attributed health harms was moderate in both groups (6.31 ± 2.14, market visitors; 6.30 ± 1.64, bus passengers) (Table 2). Also, we found that age and education level showed significant associations with awareness regarding the law of smoking restrictions in public places (Table 3), while age, education level, and religion had significant associations with the knowledge about SHS-attributed health harms (Table 4).

**Table 3:** Associations between demographic factors and study variables with knowledge about the legislated law on the smoking-ban in public places among the study participants in Meung district of Songkhla Province, Thailand, November 2021– September 2022 (N=330)

*Factors*	*Categories*	*Knowledge scores about the legislated law* *on the smoking-ban in public places^§^, n (%)*			*Total* *n (%)*	*χ^2^*	*p*
		*Low*	*Medium*	*High*			
**Sex**	Male	51 (19.2)	14 (25.0)	1 (11.1)	66 (20.0)	1.411	0.493
	Female	214 (80.8)	42 (75.0)	8 (88.9)	264 (80.0)		
**Age** (years)	<40	108 (40.8)	39 (69.6)	7 (77.8)	154 (46.7)	19.099	<0.0001*
	≥40	157 (59.2)	17 (30.4)	2 (22.2)	176 (53.3)		
**Education level**	Primary school	62 (23.4)	3 (5.4)	0 (0)	65 (19.7)	36.720	<0.0001*
	Secondary school	135 (50.9)	21 (37.5)	1 (11.1)	157 (47.6)		
	Bachelor’s or higher	68 (25.7)	32 (57.1)	8 (88.9)	108 (32.7)		
**Religion**	Buddhism	221 (83.4)	50 (89.3)	9 (100.0)	280 (84.8)	2.900	0.235
	Islam	44 (16.6)	6 (10.7)	0 (0.0)	50 (15.2)		
**Current smoking status**	Non-smoking	236 (89.1)	50 (89.3)	8 (88.9)	294 (89.1)	0.003	0.929
	Smoking	29 (10.9)	6 (10.7)	1 (11.1)	36 (10.9)		
**Total**		265 (80.3)	56 (17.0)	9 (2.7)	330 (100)		

§ Scores ≤6.00, low; 6.01–7.99, medium; and ≥8.00: high.

* Statistical significance at p<0.05.

**Table 4:** Associations between demographic factors and study variables with knowledge about secondhand smoke-attributed health harms among the study participants in Meung district of Songkhla Province, Thailand, November 2021– September 2022 (N=330)

*Factors*	*Categories*	*Knowledge scores about secondhand smokeattributed health harms^§^, n (%)*			*Total* *n (%)*	*χ^2^*	*p*
		*Low*	*Medium*	*High*			
**Sex**	Male	19 (17.9)	24 (19.2)	23 (23.2)	66 (20.0)	0.982	0.612
	Female	87 (82.1)	101 (80.8)	76 (76.8)	264 (80.0)		
**Age** (years)	<40	37 (34.9)	74 (59.2)	43 (43.4)	154 (46.7)	14.196	0.001*
	≥40	69 (65.1)	51 (40.8)	56 (56.6)	176 (53.3)		
**Education level**	Primary school	42 (39.6)	14 (11.2)	9 (9.1)	65 (19.7)	51.245	<0.001*
	Secondary school	41 (38.7)	75 (60.0)	41 (41.4)	157 (47.6)		
	Bachelor’s or higher	23 (21.7)	36 (28.8)	49 (49.5)	108 (32.7)		
**Religion**	Buddhism	77 (72.6)	112 (89.6)	91 (91.9)	280 (84.8)	18.332	<0.001*
	Islam	29 (27.4)	13 (10.4)	8 (8.1)	50 (15.2)		
**Current smoking status**	Non-smoking	95 (89.6)	108 (86.4)	91 (91.9)	294 (89.1)	1.777	0.411
	Smoking	11 (10.4)	17 (13.6)	8 (8.1)	36 (10.9)		
**Total**		106 (32.1)	125 (37.9)	99 (30.0)	330 (100)		

§ Scores ≤6.00, low; 6.01–7.99, medium; and ≥8.00: high.

* Statistical significance at p<0.05.

Based on the TPC Act 2017, the Songkhla Provincial TPC Committee was set up and composed of various local governmental agencies, such as provincial healthcare offices, police departments, provincial attorneys, academic institutional networks, provincial excise offices, and the Songkhla City Administration Office. The comments concerning the feasibility of applying the smoking restriction law in the province from the in-depth interviews were collected (see Supplementary file) and presented in the form of a thematic analysis (Table 5).

**Table 5:** Thematic analysis of the data from the in-depth interviews of the local governmental officers who worked on secondhand smoke control in Meung district of Songkhla Province, Thailand, November 2021–September 2022 (N=20)

Themes	Codes
**A. Government Office sector**	
**Policy endorsement and implementation**	National tobacco control policy was affirmed by the legislation of the TPC Act 2017 endorsed nationwide. The TPC policies were transferred to provincial and succeeded to district levels over Thailand. Provincial tobacco control committees were set up from multidisciplinary stakeholders (provincial governmental agencies) to conduct and supervise the tobacco control actions in the province following the national tobacco control policies based on the TPC Act 2017. Some relevant provincial governmental agencies did not put their full effort into provincial tobacco control actions due to lack of knowledge and understanding of their responsibilities expected on the issue.
**Feasibility and limitations of policy implementation**	Irregular and low frequency provincial tobacco control committee meetings for supervision caused the assignments to the individual provincial governmental agencies to be unclear. Limitations of manpower and budget to do the tobacco control regulation by policemen or metropolitan police. The responsible duties or responses to ensure public safety were more urgent than tobacco control actions. No visible ‘No smoking’ signs and ‘5000 THB fine for those smoking in this area’ were adequately presented in the smoking-restricted zones. No one who violated the smoking regulation was really fined. Some officers themselves were active smokers, which would make them reluctant to fine the smoking violators.
**B. Public sector**	
**Awareness of TPC Act 2017 endorsement, SHS experiences and reactions**	**Market visitors** The goods transporters working in the markets, the food sellers and buyers frequently smoked. No ‘No smoking’ or ‘5000 THB fine for smoking in this area’ signs were posted at the market entrance, exit and at easily visible sites in the marketplaces. The people working in the markets and some market visitors did not know that marketplaces were smoking prohibition areas. Tobacco smell was usually experienced and discarded cigarette butts were frequently seen in the markets, although no smokers were seen. Some people did not know that they could report the smoking violation event to the officers using online contact. Most people avoided to report smoking violations to the officers to avoid arguments with the smokers. They chose only to move far away from the smokers. **Public vehicle passengers** The bus, ‘songthaew’, and motorcycle taxi drivers were the common smokers in the public transport vehicles. The drivers were unaware of the smoking prohibition on the public vehicles and SHS-attributed health harms. The drivers misunderstood that the shielding glass separating the driver from the passenger section was effective enough in prevention of tobacco smoke to float to the passengers. The passengers on the public vehicles who were being exposed to tobacco smoke usually had no reaction to the violation. They sat quietly until they got off the vehicles when they arrived at their destinations. A portion of passengers did not know that the smoking violation was a legal fault that could be reported and the violator would be fined by the local authorized officers. For personal safety reasons, the passengers who experienced smoking violations decided not to report the events to the officers.

## DISCUSSION

Our study found that the reported percentage of visible smokers was lower than that of experiencing tobacco smell in the marketplaces (30.0% vs 49.8%) and public bus terminals (20.7% vs 45.5%). This implies that the number of smokers in both places is possibly higher than that which is visible, although participant report bias is possible. Age, level of education, and religion were significantly associated with the knowledge of SHS-attributed health harms. Meanwhile, age and education level were associated with the awareness of smoking-free areas following the TPC Act 2007. We selected marketplaces and regular public transportation systems in this province as study areas because the goods transporters in the markets and the drivers are commonly active smokers and smoking-ban enforcement was low in both places. The workers or laborers in Thailand habitually smoke. Their hard work, low income, and low literacy possibly cause them to be unaware of smoking harms and SHS-attributed health harms to non-smokers. Therefore, they were the major sources of SHS generation in the study places. A recent study on the people exposed to SHS reported that besides males, individuals who had lower education level, household incomes, or awareness of the public smoking-ban regulations had a higher percentage of SHS exposure in public places where they are commonly present for working or living^10^. A study on SHS exposure in 15 low- and middle-income countries (7 of them were in Asia, including Thailand) supports that people of low socioeconomic status have a higher risk of SHS exposure, especially where smoking ban regulations are poorly enforced^11^. To improve knowledge, attitudes, and the appropriate practices on SHS exposure in public places, effective health education programs to enhance health literacy about SHS-attributed harms should be emphasized^12^. Therefore, both active smokers and SHS-exposed non-smoker people should be intensively provided with knowledge about SHS-attributed health harms. In the meantime, legal stringency to prohibit smoking in public places has to be enforced to protect non-smokers from SHS exposure. The obvious declining prevalence of SHS exposure can be partially attributed to changes in a nation’s tobacco policy and early legislated smoking controls^13^.

Marketplaces and public transport vehicles, as well as their stops or terminals, were the sites where local people commonly congregated to use the facilities for daily living. Notably, the laborers who transported goods in the markets (52.1%) and the public vehicle drivers (80%) comprised the largest portion of smokers at the individual sites. To avoid the occurrence of an argument with the smokers, most of the people who were exposed to SHS chose to leave the places where the smokers were without action, or they protected themselves from any SHS exposure as best as possible (86.9%). Only a small number of the respondents from the marketplaces (10%) and the bus passengers (3.3%) intended to report the events to the provincial authorities. We thought that these reactions were possibly caused by the compromising manner in Thai culture. From the point of view of the market transport workers and drivers, it could be expected that they were not aware of the health complications of smoking, as well as the smoking-free zone regulations in their working areas. Based on the personal talk on this issue, the smokers themselves simply believed that the cigarette smoke commonly floated up into the open spaces or out of the windows of the vehicles, and that the shielding glass, separating the driver’s section and the passenger seats, could prevent the tobacco smoke from reaching the passengers. Therefore, correct understanding and practical guidelines should be provided and enhanced for smokers.

Various health measures and smoking cessation campaigns have been launched in Thailand since the founding of the ASHT in 1985, for example, the warning messages of tobacco-attributed health harms on cigarette packs, legally restricted cigarette purchasing among people under 18 years of age, and prohibiting cigarette advertisements on TV programs, online media, in public places, and sports stadiums, etc. Despite a favorable reduction in smoking prevalence, SHS exposure in Thailand remains in places with lower surveillance for smoking control^4,14,15^. Encouragement of complying with the endorsed smoking-free areas, along with strict smoking controls, has been undertaken in Thailand by the Ministry of Public Health and non-governmental organizations, including ASHT. It was not until 2017 that the TPC Act authorized several governmental measures to control the tobacco business chain and create smoking-free areas in many public places across the country. The actions comply with Article 20 of the WHO FCTC, in which the adverse health effects of SHS exposure on non-smokers are the focus, and comprehensive control measures regarding smoking in public places are advocated^2^.

Considering the implementation of top-down tobacco control national policies in the province, we found that some provincial official agencies involved in the tobacco control loop had limited understanding regarding their roles and responsibilities, as authorized by the law. This caused non-collaborative implementations of tobacco control measures in the province. Moreover, because of the limitations of manpower, budget, and the high routine workloads of many provincial governmental offices, the tobacco control measures undertaken in the province were less effective. Therefore, practical methods applied from the national policy, well-assigned responsibility, and a reformed collaboration of the provincial agencies to harmonize smoking regulations, particularly SHS exposure control, are crucial.

We believe that SHS exposure control in the province requires the incorporation of multidisciplinary provincial agencies, both governmental and non-governmental. While legal regulations, which would cost considerable monetary and human resources, might induce resistance or challenges against the smoking-ban regulations, the engagement of provincial people should be encouraged to facilitate legal action. Although comprehensive smoking legal restrictions in workplaces or public venues were previously reported as the most effective method in the elimination of SHS exposure among non-smokers^16-19^, we think that strong public motivation, awareness, and participation in eliminating SHS exposure are more likely to create enforcement of the legal regulations.

While most of the early SHS studies focused on households, workplaces, schools, and social venues, we selected the marketplaces and public transportation systems as the study settings because they were the places with a higher risk but lower recognition of SHS exposure in the province and across Thailand. Also, the available studies in these settings were very limited.

### Limitations

The study has some limitations, which include the small sample size, cross-sectional design, and non-randomized study samples. Moreover, due to possible response bias, social desirability bias, and no format regression analyses to control for potential covariates, this study has a low power for generalization.

## CONCLUSIONS

The knowledge about SHS-attributed health harms was not ideal, while awareness about legal restriction on SHS exposure was also limited. Age, education level and religion were significant associated factors. The transport workers in fresh-food markets and the drivers of a public bus or a high-roofed pickup taxi, were the active smokers generating secondhand smoke. The collaboration on the legal regulations among the provincial official agencies was weak. Therefore, SHS control in this province remained below expectations. Future research should focus on encouraging public engagement and designing a comprehensive collaboration between the public and local governmental sectors for effective SHS exposure control.

## Data Availability

The data supporting this research can be found in the Supplementary file.
